# Omicron BA.1 breakthrough infections in inactivated COVID-19 vaccine recipients induced distinct pattern of antibody and T cell responses to different Omicron sublineages

**DOI:** 10.1080/22221751.2023.2202263

**Published:** 2023-05-01

**Authors:** Li Guo, Qiao Zhang, Jingchuan Zhong, Lan Chen, Wentao Jiang, Tingxuan Huang, Yanan Li, Yin Zhang, Liuhui Xu, Xinming Wang, Yan Xiao, Ying Wang, Xiaojing Dong, Tao Dong, Yanchun Peng, Biao Zhang, Yan Xie, Hongmei Gao, Zhongyang Shen, Lili Ren, Tao Cheng, Jianwei Wang

**Affiliations:** a National Health Commission Key Laboratory of Systems Biology of Pathogens and Christophe Mérieux Laboratory, Institute of Pathogen Biology, Chinese Academy of Medical Sciences & Peking Union Medical College, Beijing, People’s Republic of China; b Haihe Laboratory of Cell Ecosystem, Tianjin, People’s Republic of China; c Key Laboratory of Respiratory Disease Pathogenomics, Chinese Academy of Medical Sciences, Beijing, People’s Republic of China; d Organ Transplant Center, Tianjin First Center Hospital, Tianjin, People’s Republic of China; e Laboratory of Molecular and Treatment of Liver Cancer, Tianjin First Center Hospital, Tianjin, People’s Republic of China; f Research Institute of Transplant Medicine, Nankai University, Tianjin, People’s Republic of China; g Department of Respiratory and Critical Care Medicine, West China Hospital, Sichuan University, Chengdu, People’s Republic of China; h Chinese Academy of Medical Sciences Oxford Institute, Nuffield Department of Medicine, Oxford, United Kingdom; i MRC Human Immunology Unit, MRC Weatherall Institute of Medicine, Oxford University, Oxford, United Kingdom; j Haihe Laboratory of Cell Ecosystem, State Key Laboratory of Experimental Hematology, National Clinical Research Center for Blood Diseases, Institute of Hematology & Blood Diseases Hospital, Chinese Academy of Medical Sciences & Peking Union Medical College, Tianjin, People’s Republic of China; k Tianjin Institutes of Health Science, Tianjin, People’s Republic of China; l Intensive Care Unit, Emergency Medical Research Institute, Tianjin First Center Hospital, Tianjin, People’s Republic of China; m NHC Key Laboratory for Critical Care Medicine, Tianjin First Center Hospital, Tianjin, People’s Republic of China

**Keywords:** SARS-CoV-2, Omicron sublineages, breakthrough infection, humoral immune responses, cellular immune responses

## Abstract

The adaptive immunity against SARS-CoV-2 prototype strain and Omicron sublineages induced by BA.1 breakthrough infection in vaccinees of inactivated COVID-19 vaccines have not been well characterized. Here, we report that BA.1 breakthrough infection induced mucosal sIgA and resulted in higher IgG titers against prototype strain and Omicron sublineages in vaccinees than in vaccine naïve-infected individuals. BA.1 breakthrough infection boosted antibody-dependent cellular cytotoxicity and antibody-dependent cellular phagocytosis to prototype strain and BA.1, BA.1.1, BA.2, BA.2.12.1, and BA.2.75 but not BA.4/5 and induced neutralization against prototype strain and BA.1, BA.1.1, BA.2, BA.2.12.1, BA.2.75, and BA.4/5 but not BF.7, BQ.1, and XBB. In total, BA.1 breakthrough infection individuals produced less extensive sIgA, plasma IgG and NAb responses against Omicron sublineages compared with those against prototype strain. Further, BA.1 breakthrough infection induced recall B cell response to prototype strain and Omicron variant, primarily targeting memory B cells producing conserved epitopes. Memory T cell responses against Omicron is largely preserved. Individuals with vaccine booster did not induce more beneficial immune responses to Omicron sublineages upon BA.1 breakthrough infection than those with primary vaccine dose only. The breakthrough infection individuals produced stronger adaptive immunity than those of inactivated vaccine-healthy individuals. These data have important implications for understanding the vaccine effectiveness and adaptive immunity to breakthrough infection in individuals fully immunized with inactivated vaccines. Omicron sublineages, especially for those emerged after BA.4/5 strain, evade NAb responses induced by BA.1 breakthrough infection. It is urgent to optimize the vaccine immunogen design and formulations to SARS-CoV-2 variants.

## Introduction

The SARS-CoV-2’s genome has rapidly evolved since it emerged in late 2019. Within 3 years, numerous mutations in the SARS-CoV-2 genome relevant to its fitness have resulted in a series of variants of concern (VOCs), such as Alpha, Beta, Gamma, Delta, and Omicron [1]. The mutations in VOCs are primary in the receptor-binding domain (RBD) and N-terminal domain (NTD) targeted by potent NAbs. After the initial wave of infection by the BA.1 strain, over 400 Omicron sublineages have been identified [2–5]. Multiple COVID-19 vaccine platforms have been developed and deployed to control the COVID-19 pandemic. As of Dec 23, 2022, a cumulative total of 3,469 million doses of COVID-19 vaccine have been inoculated in China, most of which are inactivated vaccines [6]. These inactivated vaccines substantial contribute to preventing severe disease and hospitalizations in many regions of the world [7, 8].

Compared with the SARS-CoV-2 prototype strain, the BA.1 strain harbor multiple mutations in the spike protein [2]. These mutations cause the high transmissibility and immune evasion from existing SARS-CoV-2 NAbs elicited by prior natural infection or vaccination, contributing to high levels of vaccine breakthrough infections [9]. Omicron sublineages continue to emerge. BA.2 and BA.1.1, highly related but contain unique mutations in the spike protein, have been described [10]. BA.4 and BA.5 (referred to as BA.4/5 here after) have become dominant Omicron sublineages since first reported in early April 2022. The prevalence of the BA.2.75 is increasing in Indian and BA.2.75 has been detected in at least 58 countries as of Oct 17, 2022 [11]. BF.7, BQ.1, and XBB sublineages are spreading (https://www.cdc.gov/). However, how the adaptive immunity induced by inactivated vaccines evolves through iteration with the infection of the Omicron variant remains poorly understood.

Here, we characterize the antibody, memory B cell, and memory T cell responses against Omicron sublineages in individuals who experienced BA.1 breakthrough infection after double or triple-vaccinated with BBIBP-CorV or CoronaVac inactivated COVID-19 vaccine (inactivated vaccine-infected hereafter). We included BA.1 infection individuals without received COVID-19 vaccine (vaccine naïve-infected), uninfected individuals who had received inactivated COVID-19 vaccine (inactivated vaccine-healthy) and without received COVID-19 vaccine (vaccine naïve-healthy) as controls.

## Materials and methods

### Study design and participants

BA.1 first emerged on January 8, 2022, in Tianjin, China and caused a large wave of infections [12]. The objective of this study was to investigate the humoral and cellular immune responses to BA.1 breakthrough infection in individuals vaccinated with inactivated vaccine (Sinopharm BBIBP-CorV or Sinovac CoronaVac inactivated COVID-19 vaccine in this study). In this cohort study, we recruited vaccine naïve individuals with BA.1 infection (vaccine naïve-infected) and vaccinated individuals with subsequent BA.1 breakthrough infection (inactivated vaccine-infected) at Tianjin First Central Hospital, Tianjin, China, between Feb 11, 2022 and Feb 23, 2022. Participants had no symptom at the time of blood collection. All participants had no documented history of SARS-CoV-2 infection prior to vaccination. We also included inactivated vaccine-healthy individuals and vaccine naïve-healthy individual as control in this study. Written informed consent was obtained from each individual. The study was approved by the Institutional Review Boards of Tianjin First Central Hospital (2022N151KY).

### Inclusion and exclusion criteria

Inclusion criteria for breakthrough infection individuals:

1. those who were at least 18 years old.
2. those who were laboratory confirmed COVID-19 by RT–PCR in BA.1 wave of infections.
3. those who received at least two doses of inactivated vaccine for breakthrough infection.

Exclusion criteria

1. those who had HIV, active hepatitis, or active tuberculosis infection.
2. those who have had a bone marrow transplant or are receiving chemoradiotherapy for cancer
3. those who are taking immunosuppressants
4. those who had immune dysfunction caused by genetic defects.
5. those who have received stem cell therapy within six months.
6. those who had a history of congenital heart disease.
7. those who had received SARS-CoV-2 monoclonal antibody therapy

### Throat swab samples collected

Throat swab (TS) samples maintained in 3 mL viral transport medium (VTM) were collected by clinicians and stored at −80°C until use.

### Plasma and PBMC isolation

20 mL venous blood was collected from participants and processed within 12 h to isolate plasma and peripheral blood mononuclear cells (PBMCs). Plasma was separated by centrifugation at 300 g for 10 min and stored at −80°C until testing. PBMCs were isolated from blood using Ficoll-Paque PLUS (GE Healthcare, Chicago, IL) according to the manufacturer’s instructions. Isolated PBMCs were frozen in 90% heat-inactivated fetal bovine serum (FBS, Hyclone, Northbrook, IL) supplemented with 10% DMSO (Sigma-Aldrich, St. Louis, MO, USA), and stored in liquid nitrogen before analysis.

### Enzyme-linked immunosorbent assay (ELISA)

Titers of plasma IgG and mucosal sIgA against the spike protein (S) of SARS-CoV-2 prototype strain and BA.1, BA.1.1, BA.2, BA.2.12.1, BA.2.75, and BA.4/5 were evaluated using the enzyme-linked immunosorbent assay (ELISA) as previously reported [13]. The optimal coating concentration of S protein of SARS-CoV-2 prototype strain and Omicron sublineages (ACROBiosystems, Beijing, China) were 50 ng. The optimal plasma dilutions were 1/400. The second antibodies were horseradish peroxidase conjugated goat anti-human Fc specific polyclonal IgG or goat rabbit anti-human α chain specific polyclonal IgA (Sigma Aldrich, St Louis, MO, USA), respectively.

### Antibody-dependent cellular cytotoxicity (ADCC) and antibody-dependent cellular phagocytosis (ADCP) activity bioassays

ADCC bioassay effector cells (Jurkat-FcγRIIIa-V158-NFAT-Luc) and ADCP bioassay effector cells (Jurkat-FcγRIIa-H131-NFAT-Luc) were obtained from Vazyme (Nanjing, China) and cultured in RPMI 1640 medium with 10% FBS. All cells were cultured at 37°C with 5% CO2.

The ADCC or ADCP reporter assay was performed in accordance with the manufacturer's instructions. The assays were performed using 293T-spike cells that expressed SARS-CoV-2 S protein as target cells. To obtain target cells, plasmids expressing viral S protein, pCAGGS-prototype-S, pCAGGS-BA.1-S, pCAGGS-BA.1.1-S, pCAGGS-BA.2-S, pCAGGS-BA.2.12.1-S, pCAGGS-BA.2.75-S, and pCAGGS-BA.4/5-S, were transfected into 293 T cells for 24 h, respectively. For all reporter assays, target cells (1.25 × 10^4^ per well) were incubated with plasma samples at 1/200 dilution. Effector cells (7.5 × 10^4^ per well) were then added to each well. After incubation at 37 °C for 24 h, 75 μl of Bright-Lite Reagent (Vazyme) were added to the wells and incubated for 5–10 min, and the relative light unit (RLU) was detected with Modulus II (Promega, CA, USA). The fold of induction was calculated as follows: RLU (induced − background)/RLU (no serum control − background).

### Live virus microneutralization assay

NAbs were assessed on Vero cells (ATCC, Manassas, VA, CCL-81) infected with the SARS-CoV-2 prototype strain (IPBCAMS-WH-01/2019, no. EPI_ISL_402123) and BA.1 and BA.5, which were isolated from respiratory tract samples from COVID-19 patients in a biosafety level 3 laboratory using a microneutralization assay as previously reported [13]. The cut-off for a positive NAb titre was 1/10. When plasma sample has no neutralizing activity, the NAb titer is defined as 1/5.

### Pseudovirus microneutralization assays

Pseudovirus of prototype strains, and BA.1, BA.1.1, BA.2, BA2.12.1, BA2.75, BA.4/5, BF.7, BQ.1, and XBB were generated by co-transfection of 293 T cells with pLenti-Luciferase, psPAX2 and viral S protein expression plasmids, pCAGGS-prototype-S, pCAGGS-BA.1-S, pCAGGS-BA.1.1-S, pCAGGS-BA.2-S, pCAGGS-BA.2.12.1-S, pCAGGS-BA.2.75-S, pCAGGS-BA.4/5-S, pCAGGS-BF.7-S, pCAGGS-BQ.1-S, and pCAGGS-XBB-S. Neutralization assays were performed as follows. Briefly, 293T-ACE2 cells were seeded in a 96-well plate at a concentration of 1 × 10^4^ cells per well in 100 μL of DMEM with 10% FBS and cultured for 12–16 h. Fivefold serially diluted plasma starting at 1/40 from recovered patients were incubated with SARS-CoV-2 pseudotyped virus for 1 h at 37 °C. The mixture was added to 293T-ACE2 cells. After 48 h post-infection, luciferase activity was measured with a luciferase assay system (Promega, E1501) on a multifunctional microplate reader SpectraMax M5. The titers of SARS-CoV-2–specific NAb were calculated as a 50% half-maximal inhibitory does (ID50) and expressed as the dilution of plasma that resulted in a 50% reduction of luciferase luminescence compared with virus control in single-round pseudovirus infection assay. The cut-off for a positive NAb titer was 1/80.

### Detection of prototype SARS-CoV-2–specific and Omicron BA.1 and BA.4/5 RBD-specific memory B cells

B cells were enriched using Pan B Cell Isolation Kit (Miltenyi Biotec, Bergisch Gladbach, Germany) from PBMC according to the manufacturer’s protocol. The prototype SARS-CoV-2 S- and RBD-specific B cells, and BA.1 and BA.4/5 RBD-specific B cells were detected using biotinylated proteins in combination with different streptavidin-fluorophore conjugates. All reagents are listed in Table S1. Biotinylated proteins were multimerized with fluorescently labeled streptavidin (molar ratio ∼4:1). After multimerization individually, enriched Pan B cells were first stained with Zombie NIR™ Fixable Viability Kit for 15 min at room temperature. Cells were then washed and stained with antigen probe master mix, APC-anti-human CD19, BV421-anti-human IgD, PerCP/Cy5.5-anti-human IgG, BV510-anti-human CD27, PE/Cy7-anti-human CD38 for 40 min at 4°C. After surface stain, cells were washed and fixed in 1% paraformaldehyde (PFA) for 20 min at 4°C. Gating strategy for flow cytometry analysis were shown in Figure S1. Antigen-specific gates for B cell probe assays were set based on healthy donors stained without antigen probes and were kept the same for all experimental runs.

### Peptide synthesis

The 15- to 18-mer peptides that overlap by 10 amino acid residues and span the S protein of BA.1 and BA.4/5 were synthesized, respectively. All peptides were synthesised by Sangon Biotech (purity >90%; Sangon Biotech, Shanghai, China).

### Activation induced marker (AIM) assay

SARS-CoV-2–specific T cells were detected using an activation induced marker assay. All reagents are listed in Table S1. Cryopreserved PBMCs were thawed, washed, resuspended at 5 × 10^6^/ml in RPMI supplemented with 10% FBS, 2 mM L-glutamine, 100 U/mL penicillin, and 100 mg/mL streptomycin (R10). After rested for 3 h at 37°C, CD40 blocking antibody was added to cultures for 15 min before stimulation. Cells were then stimulated for 18 h with BA.1 and BA.4/5 peptide megapools, respectively, at a final concentration of 2 μg/mL. No peptide stimulation was used as a negative control. PE anti-CD40L, FITC anti-CD107a, PE-Dazzle 594 anti-CCR7, BV605 anti-CXCR3, APC-Cy7 anti-CXCR5 were added to the culture along with peptide pools. After 18 h, cells were stained with BD Horizon Fixable Viability Stain 510 and Fc receptor blocking solution (Human TruStain FcX, BioLegend). Surface staining for 30 min was then performed with antibodies against PerCP-Cy5.5 anti-CD3, BV650 anti-CD4, PE-Cy7 anti-CD8, BV785 anti-CD45RA, PE anti-CD40L, APC anti-OX40, and BV421 anti-4-1BB. Specific cytokine responses were calculated by subtracting the background activation. AIM + T cells were defined by an analysis identifying cells coexpressing at least two of four markers: CD40L, CD107a, OX40, 4-1BB. All samples were acquired on a BD LSRFortessa (BD Biosciences) flow cytometer and analyzed using FCS Express 7 (De NOVO software, Pasadena, CA).

### Intracellular cytokine staining (ICS)

PBMC were expanded in vitro as previously described [13]. Expanded PBMCs were incubated with pooled peptides at a final concentration of 10 μg/mL for 1 h, and then with brefeldin A and monensin for an additional 5 h. Dead cells were labeled using BD Horizon Fixable Viability Stain 510 (BD Biosciences). Surface staining was performed using PerCP-Cy5.5-anti-CD3, BV650-anti-human CD4, PE-Cy7-anti-human CD8 (Biolegend). After incubation with fixation/ permeabilization stain buffer (Invitrogen, Carlsbad, CA), cells were immunostained using BV421-anti-IFNγ, BV711-anti-TNFα, APC-anti-IL-2 (Biolegend). Information about reagents used for intracellular cytokine staining (ICS) are listed in Table S1. No peptide stimulation was used as a negative control for each condition. Specific cytokine responses were calculated by subtracting the background activation before further analysis. T cells exposed to PMA/ionomycin served as positive controls. All samples were acquired on a BD LSRFortessa (BD Biosciences) flow cytometer and analyzed using FCS Express 7 (De NOVO software, Pasadena, CA). Single-stained CompBeads (BD Biosciences) or single-stained PBMCs were used for compensation. Unstained PBMCs were used for assessing autofluorescence.

### Multiparametric correlation matrix analysis

Correlograms plotting the Spearman rank correlation coefficient (r) between all paired parameters were created with the scipy package (v1.7.3) running in Spyder (5.3.3). Spearman rank two-tailed *p* values were calculated using scipy.stats.spearman and graphed based on *p < 0.05, **p < 0.01, ***p < 0.001, ****p < 0.0001.

### Statistical analysis

Demographic characteristics of patients presented as median (IQR) for continuous variables and expressed as absolute values along with percentages for categorical variables. The comparison of seropositivity of IgG and NAbs was done with χ² test, or Fisher's exact test when appropriate. Single comparisons between other metrics were performed using the Mann–Whitney U test. Multiple comparisons of antibody titers, memory B cell and memory T cell responses were performed using the Kruskal–Wallis test. Paired antibody titers, memory B cell and memory T cell responses were compared using a two-tailed Wilcoxon matched-pairs signed-rank test. Spearman correlation analysis was performed for single continuous variate correlation analyses. A two-sided *p* < 0.05 was considered to be statistically significant. All statistical analysis was conducted using GraphPad Prism 9.4 (GraphPad Software, San Diego, CA).

## Results

### Cohort and sampling

We collected blood samples and TS samples from 55 vaccinated individuals with subsequent BA.1 breakthrough infection experienced in a period of BA.1 wave infection, as well as from 5 vaccine naïve individuals with BA.1 infection (Table 1). Breakthrough infection individuals received two or three doses of inactivated vaccine. The median age was 40 years (range 18–69; interquartile range [IQR] 30–56) and 24 (43.6%) were male. The median duration since the last vaccination to breakthrough infection was 67 days (rang 10–253; IQR 38–163) and the median duration between breakthrough infection and sample collection was 26 days (range 13–35; IQR 22–29). TS samples were used to evaluate mucosal secretory IgA (sIgA). Plasma samples were used to determine the IgG titers, antibody-dependent cellular cytotoxicity (ADCC) and antibody-dependent cellular phagocytosis (ADCP) activities, and NAb titers against the prototype strain and Omicron sublineages. PBMCs were used to characterize memory B cell and memory T cell responses against the prototype strain and the Omicron variant (Table S2). Due to insufficient PBMC samples being obtained, only 42 and 16 PBMC samples from breakthrough infection patients were used to evaluate memory B and memory T cell responses, respectively.

**Table 1.** Demographic characteristics of patients in this study.

Characteristics	Total	Inactivated vaccine-infected		Vaccine naïve-infected	Inactivated vaccine-healthy	Vaccine naïve-healthy
		2-dose	3-dose			
No. of participants	90	15	40	5	15	15
Age, Median (IQR), y	38 (29–56)	38 (23–55)	42 (35–58)	29 (26–46)	51 (37–59)	29 (25–38)
Sex, n (%)						
Male	41 (45.6%)	3 (20.0%)	21 (52.5%)	1 (20%)	9 (60%)	7 (46.7)
Female	49 (54.4%)	12 (80.0%)	19 (47.5%)	4 (80%)	6 (40%)	8 (53.3)
Days after infection						
Median (IQR)	27 (22–29)	27 (25–29)	26 (22–29)	27 (27–31)	/	/
Days after vaccination						
Median (IQR)	82 (45–159)	195 (154–203)	55 (36–89)	/	35 (35–36)	/

Inactivated vaccine=BBIBP-CorV (Sinopharm) or CoronaVac (Sinovac);

Data are n (%), n/N (%), or median (IQR). IQR= interquartile range.

We obtained 15 plasma and PBMC samples from inactivated vaccine-healthy individuals. The median age was 51 years (range 31–70; IQR 37–59) and 9 (60.0%) were male. The median duration since the last vaccination to sample collection was 35 days (range 32–36; IQR 35–36). At the same time, 15 plasma samples collected before 2019 from vaccine naïve-healthy individuals were included in this study. The median age was 29 years (range 21–66; IQR 25–38) and 7 (46.7%) were male. In these healthy individual controls, plasma samples were used to determine the IgG, ADCC and ADCP, and NAb titers against the prototype strain and Omicron sublineages. PBMCs were used to characterize memory B cell and memory T cell responses (Table S2).

### Mucosal sIgA and plasma IgG responses following BA.1 breakthrough infection

**Figure 1.**
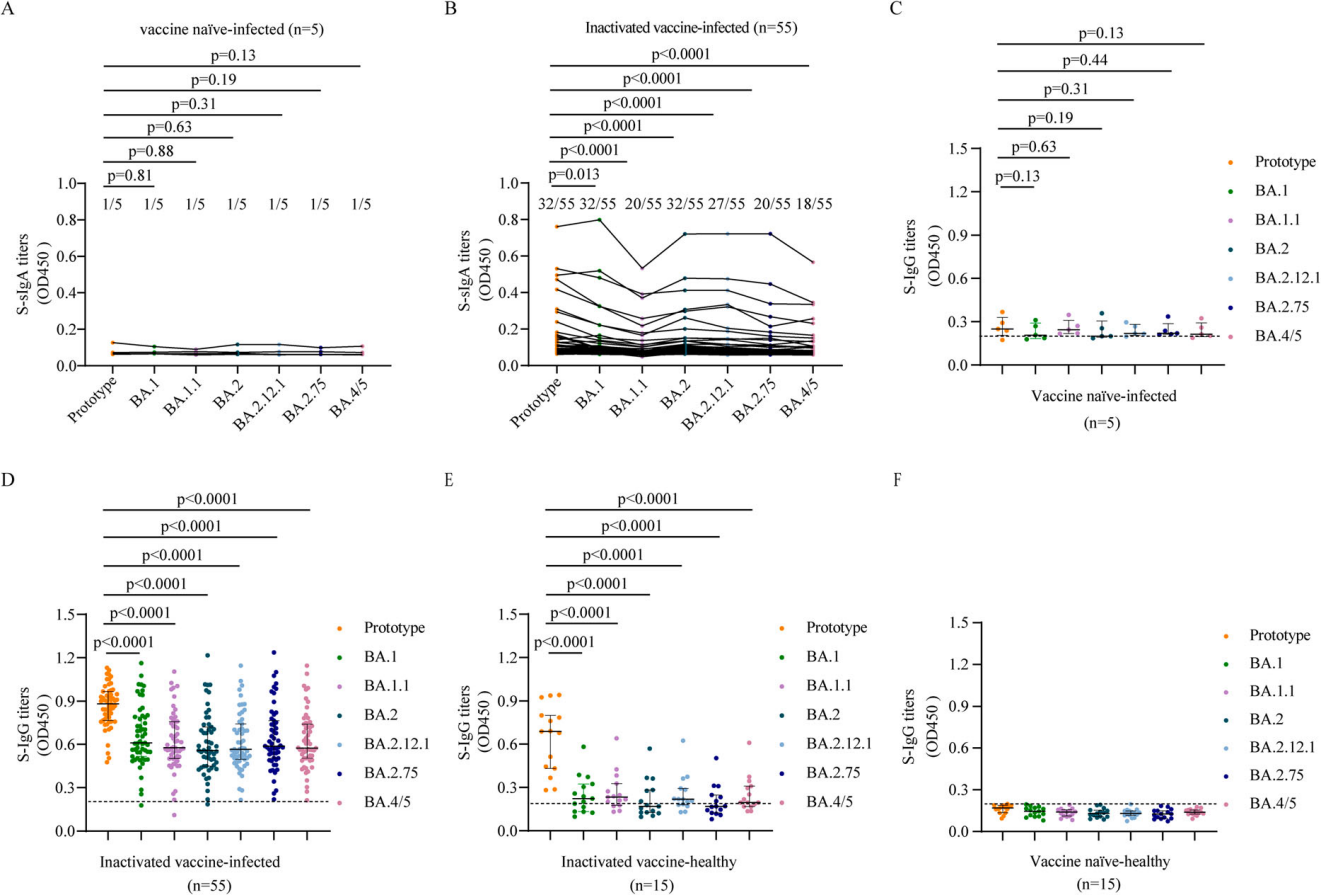
Mucosal sIgA and plasma IgG against SARS-CoV-2 prototype strain and Omicron sublineages. A–B. Mucosal S-sIgA responses against SARS-CoV-2 prototype strain and Omicron BA.1, BA.1.1, BA.2, BA.12.1, BA.2.75, and BA.4/5 induced by vaccine naïve-infected individuals (A) and inactivated vaccine-infected individuals (B). C–F. Plasma S-IgG titers against SARS-CoV-2 prototype strain and Omicron BA.1, BA.1.1, BA.2, BA.12.1, BA.2.75, and BA.4/5 induced by vaccine naïve-infected individuals (C), inactivated vaccine-infected individuals (D), inactivated vaccine-healthy individuals (E), and vaccine naïve-healthy individuals (F). Data in (C–F) are shown as the median with interquartile range (IRQ). The paired comparisons of sIgA and IgG titers were performed using two-tailed Wilcoxon matched-pairs signed-rank test.

We first evaluated TS sIgA and plasma IgG responses to the spike proteins (S) of SARS-CoV-2 prototype strain and Omicron sublineages BA.1, BA.1.1, BA.2, BA.2.12.1, BA.2.75, and BA.4/5 following breakthrough infection. We found that vaccine naïve-infected individuals did not produce effective S-sIgA (Figure 1A) and plasma S-IgG (Figure 1C) response against the prototype strain and Omicron sublineages after BA.1 infection. About 58.2% (32/55) breakthrough infection vaccinees of inactivated vaccine had detectable S-sIgA against the prototype strain, and the sIgA titres decreased in BA.1, BA.1.1, BA.2, BA.2.12.1, BA.2.75, and BA.4/5 (*p* = 0.013, *p* < 0.0001, *p* < 0.0001, *p* < 0.0001, *p* < 0.0001, *p* < 0.0001, respectively; Figure 1B). Moreover, plasma samples had significantly lower S-IgG titers to BA.1, BA.1.1, BA.2, BA.2.12.1, BA.2.75, and BA.4/5 than those to the prototype strain in breakthrough infection individuals (all *p* < 0.0001; Figure 1D). These results indicated that in individuals vaccinated with inactivated vaccine, BA.1 breakthrough infection induced diminished S-sIgA and S-IgG against Omicron sublineages than those against the prototype strain, although significantly higher than those in vaccine naïve individuals infected by BA.1.

We found that the S-IgG titers against prototype strain and Omicron sublineages were lower in inactivated vaccine-healthy individuals than those in breakthrough infection individuals (Figure 1D, 1E). But the S-IgG titers against prototype strain were significantly higher than those against Omicron sublineages in inactivated vaccine-healthy individuals (all *p* < 0.0001; Figure 1E), which was similar with the pattern in breakthrough infection individuals. While no SARS-CoV-2 specific IgG were detected in the vaccine naïve-healthy individuals (Figure 1F).

### ADCC and ADCP activities induced by BA.1 breakthrough infection

**Figure 2.**
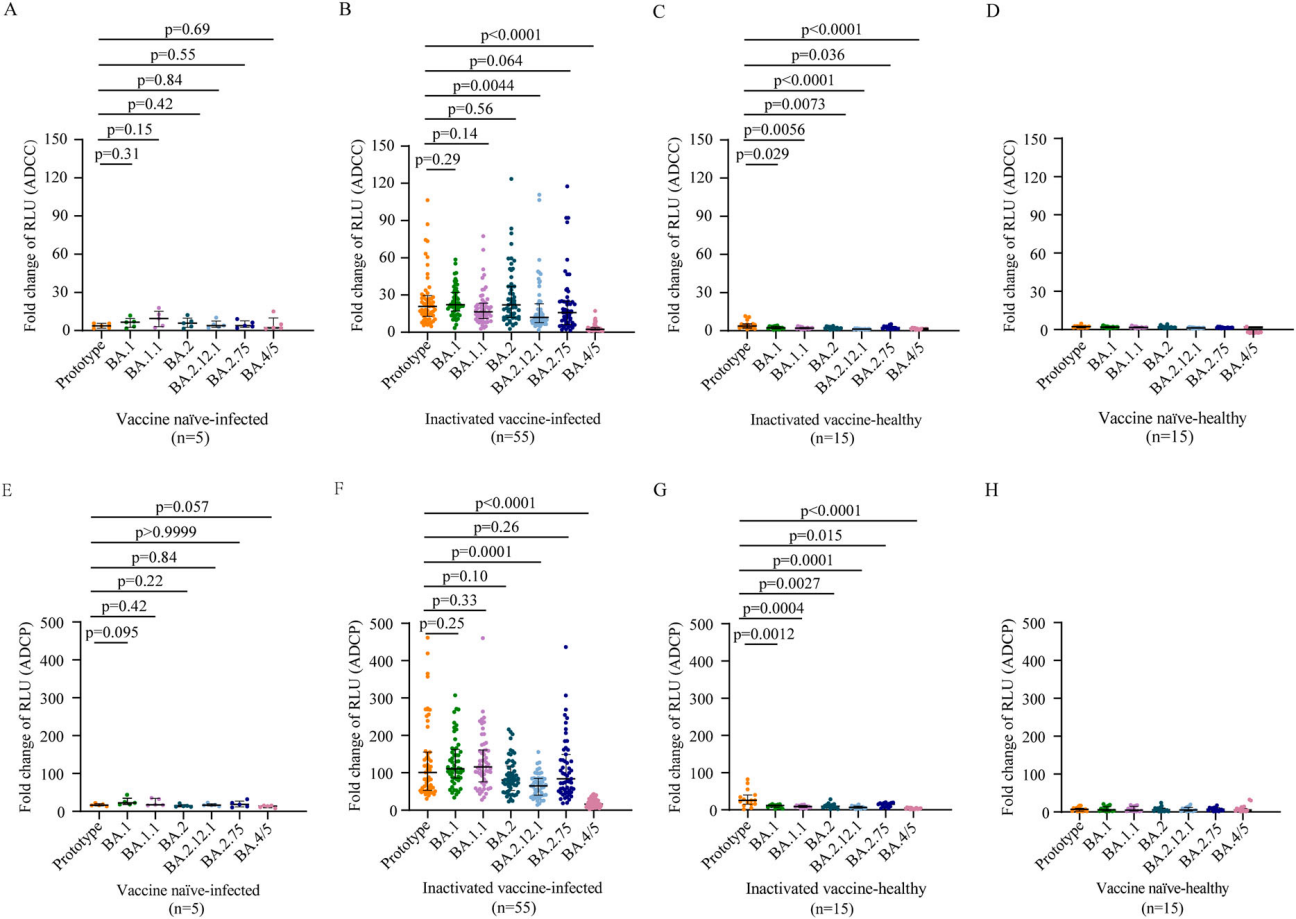
ADCC and ADCP activities against SARS-CoV-2 prototype strain and Omicron sublineages. ADCC (A–D) and ADCP (E–H) activities against SARS-CoV-2 prototype strain and Omicron BA.1, BA.1.1, BA.2, BA.12.1, BA.2.75, and BA.4/5 in vaccine naïve-infected individuals (A, E), inactivated vaccine-infected individuals (B, F), inactivated vaccine-healthy individuals (C, G), and vaccine naïve-healthy individuals (D, H). Data are shown as the median with IRQ. The paired comparisons of ADCP and ADCC activities were performed using two-tailed Wilcoxon matched-pairs signed-rank test.

Next, we evaluated ADCC and ADCP activities induced by BA.1 infection. Like the plasma S-IgG results, vaccine naïve-infected individuals did not produce effective ADCC and ADCP activities after BA.1 infection (Figure 2A, 2E). Plasma samples from BA.1 breakthrough infection individuals displayed similar ADCC activities (Fold of RLU induction: 20.8 vs 22.2, 16.4, 22.1,16.0, respectively) and ADCP activities (101.2 vs 110.8, 115.9, 81.2, 84.1, respectively) against the prototype strain and BA.1, BA.1.1, BA.2, BA.2.75. However, ADCC and ADCP activities against BA.2.12.1 (11.9 and 64.7, respectively) and BA.4/5 (2.25 and 16.2, respectively) were significantly lower than those of the prototype strain (20.8 and 101.2, respectively) (Figure 2B, 2F). Thus, BA.1 breakthrough infection individuals vaccinated with inactivated vaccine induced broad ADCC and ADCP effector functions against Omicron sublineages, except for BA.4/5.

Poor ADCC and ADCP activities against prototype strain and no ADCC and ADCP activities against Omicron sublineages were induced in inactivated vaccine-healthy individuals, respectively (Figure 2C, 2G). As respected, no ADCC and ADCP activities against prototype strain and Omicron sublineages were detected in the vaccine naïve-healthy individuals (Figure 2D, 2H).

### Neutralizing antibody responses elicited by BA.1 breakthrough infection

**Figure 3.**
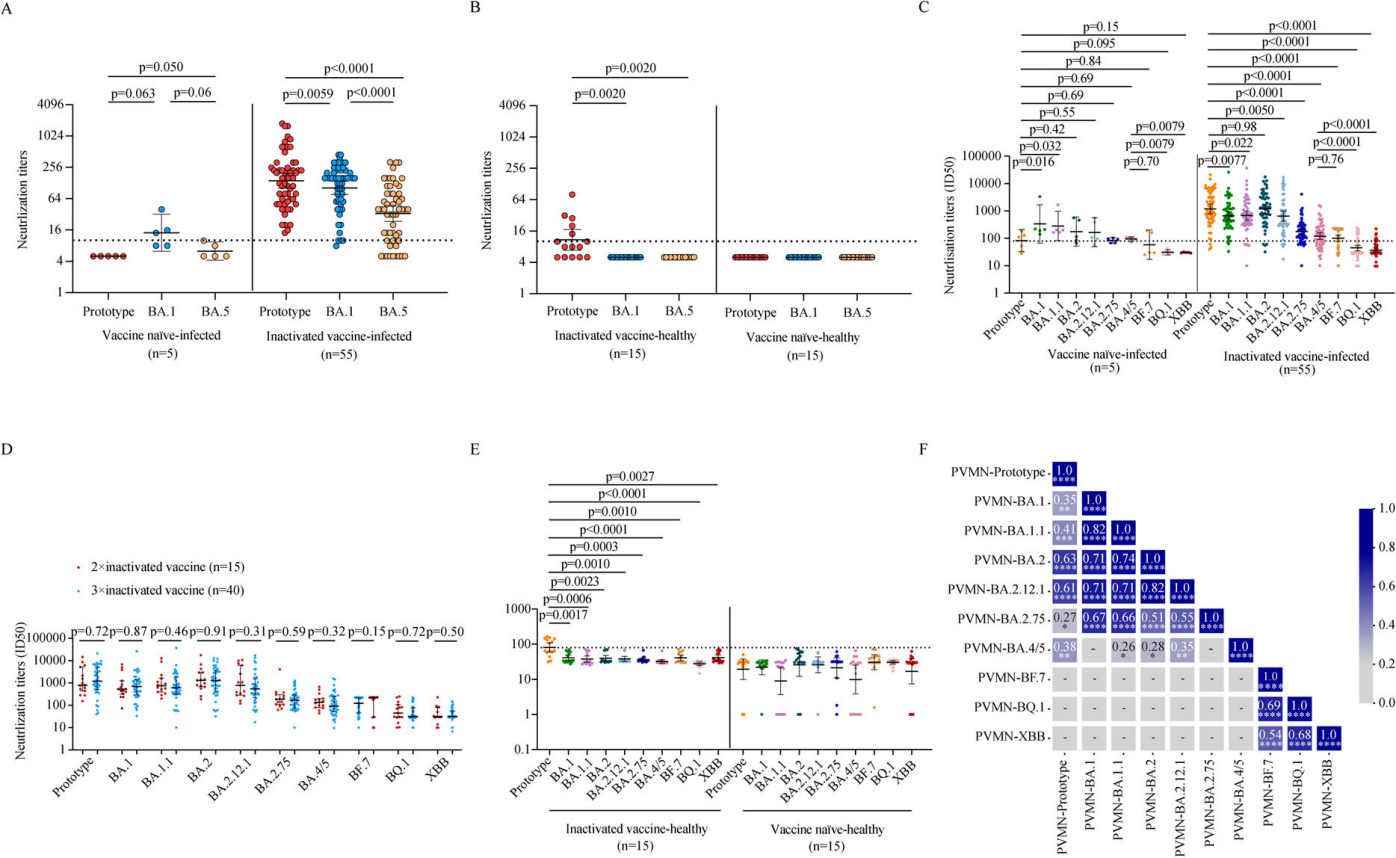
Neutralization against SARS-CoV-2 prototype strain and Omicron sublineages. A–B. Paired live virus neutralization titers against the prototype, BA.1, and BA.5 strain in vaccine naïve-infected individuals and inactivated vaccine-infected individuals (A), inactivated vaccine-healthy individuals and vaccine naïve-healthy individuals (B). C. Pairwise PVMN activities (50% half-maximal inhibitory does; ID50) against the prototype strain, and Omicron sublineages BA.1, BA.1.1, BA.2, BA. 2.12.1, BA.2.75, BA.4/5, BF.7, BQ.1, and XBB in vaccine naïve individuals with Omicron BA.1 infection and in individuals vaccinated with inactivated vaccine with BA.1 breakthrough infection. D. PVMN activities against the prototype strain and Omicron sublineages BA.1, BA.1.1, BA.2, BA.2.12.1, BA.2.75, BA.4/5, BF.7, BQ.1, and XBB among individuals with 2-dose inactivated vaccine primer, 3-dose inactivated vaccine booster, with BA.1 breakthrough infection. E. PVMN activities against the prototype strain and Omicron sublineages BA.1, BA.1.1, BA.2, BA.2.12.1, BA.2.75, BA.4/5, BF.7, BQ.1, and XBB in inactivated vaccine-healthy individuals and vaccine naïve-healthy individuals. F. Correlation matrix of PVMN activities against the prototype strain, and Omicron sublineages BA.1, BA.1.1, BA.2, BA.2.12.1, BA.2.75, BA.4/5, BF.7, BQ.1, and XBB. The dotted line in (A–C, E) denotes the cutoff value for positive neutralizing antibody titer. Solid lines in (A–E) denote the geometric mean titres (GMT) with 95% confidence interval (CI). Multiple comparisons of neutralizing antibody titers were performed using the Kruskal-Wallis test. The paired comparisons of neutralizing antibody titers were performed using two-tailed Wilcoxon matched-pairs signed-rank test. Single comparisons between 2-dose inactivated vaccine primer and 3-dose inactivated vaccine booster were performed using the Mann-Whitney U test. For the correlation analyses, correlograms plotting the Spearman rank correlation coefficient (r) between all paired parameters were created with the scipy package (v1.7.3) running in Spyder (5.3.3). *p < 0.05, **p < 0.01, ***p < 0.001, ****p < 0.0001. PVMN = pseudovirus neutralization.

We first performed a live SARS-CoV-2 microneutralization test using prototype, BA.1, BA.5 strain. Although vaccine naïve individuals infected with BA.1 had low NAb titers (GMT 14.2 [95% CI 6.3–32.0]) against BA.1 itself, they did not have cross-neutralizing activities to the prototype strain and BA.5 strain in a live SARS-CoV-2 microneutralization test (Figure 3A, left). BA.1 breakthrough infection individuals vaccinated with inactivated vaccine showed enhanced NAb activities to not only the BA.1 strain but also prototype and BA.5 strains (Figure 3A, right). However, the NAb titers against the prototype were the highest and those against BA.5 were the lowest in inactivated vaccine breakthrough infections (*p* = 0.0059, *p* < 0.0001, *p* < 0.0001, respectively) (Figure 3A, right). Only 46.7% (7/15) inactivated vaccine-healthy individuals showed NAb activities at low level (GMT 21.3 [95% CI 10.7–42.4]) against prototype strain, and none presented cross-neutralizing activities to the BA.1 and BA.5 strain (Figure 3B, left). No NAb activity against both prototype strain and omicron variants were detected in vaccine naïve-healthy individuals (Figure 3B, right).

In vaccine naïve-infected individuals, the pseudovirus microneutralization (PVMN) assay showed that BA.1 infection elicited different levels of neutralization against various Omicron sublineages: BA.1 [GMT 50% inhibitory doses (ID50), 335.4], BA.1.1 (284.1), BA.2 (177.4), BA.2.12.1 (166.2), BA.2.75 (84.5), and BA.4/5 (94.6), and induced cross-reactive NAbs against BF.7 (58.8), BQ.1 (31.1), and XBB (30.3) below the threshold level (Figure 3C, left). These data indicated that BA.1 infection did not effectively stimulate NAb cross-responsive to Omicron sublineages in vaccine naïve individuals. The PVMN assay revealed that BA.1 breakthrough infection induced broadly enhanced neutralization in individuals vaccinated with inactivated vaccine (Figure 3C, right). In BA.1 breakthrough infection individuals, compared to the NAb titers against the prototype strain (1197.0), NAb titers against BA.1, BA.1.1, BA.2.12.1 (659.9, 671.5, and 650.9, respectively) were lower (*p* = 0.0077, 0.022, 0.0050, respectively) and those against BA.2.75, BA.4/5, and BF.7 were even lower (170.4, 120.3 and 101.5, respectively; all *p* < 0.0001) (Figure 3C, right). The NAb titer against BA.2 did not differ significantly to that against the prototype (1186.0 vs 1197.0, *p* = 0.98) but there were little neutralization responses against BQ.1 and XBB strains (45.2 and 38.0, respectively). These data suggested that BA.4/5 and BA.2.75 significantly, and even more for BF.7, BQ.1, and XBB, evaded the neutralization elicited by BA.1 breakthrough infection. We found that breakthrough infection after 3-dose inactivated vaccine booster did not lead to more beneficial cross-neutralization to Omicron sublineages as compared with those after 2-dose inactivated vaccine primary immunization (all *p* > 0.05; Figure 3D). Both inactivated vaccine- and vaccine naïve-healthy individuals did not show NAb activities against Omicron sublineages in PVMN assay (Figure 3E).

Multiparametric correlation matrix analyses of inactivated vaccine-infected individuals showed that NAb titers against the prototype had stronger correlations with NAb titers against BA.2 and BA.2.12.1 than with NAb titers against BA.1, BA.1.1, BA.2.75, and BA.4/5. Moreover, NAb titers against Omicron sublineages BA.1, BA.1.1, BA.2, BA.2.12.1, BA.2.75 had strong correlations among themselves but had weak or not correlations with those against BA.4/5, BF.7, BQ.1, and XBB strain (Figure 3F). These data suggest that BA.4/5, BF.7, BQ.1, and XBB were more antigenically distant Omicron sublineages.

### Cross-reactivity of memory B cells induced by BA.1 breakthrough infection

**Figure 4.**
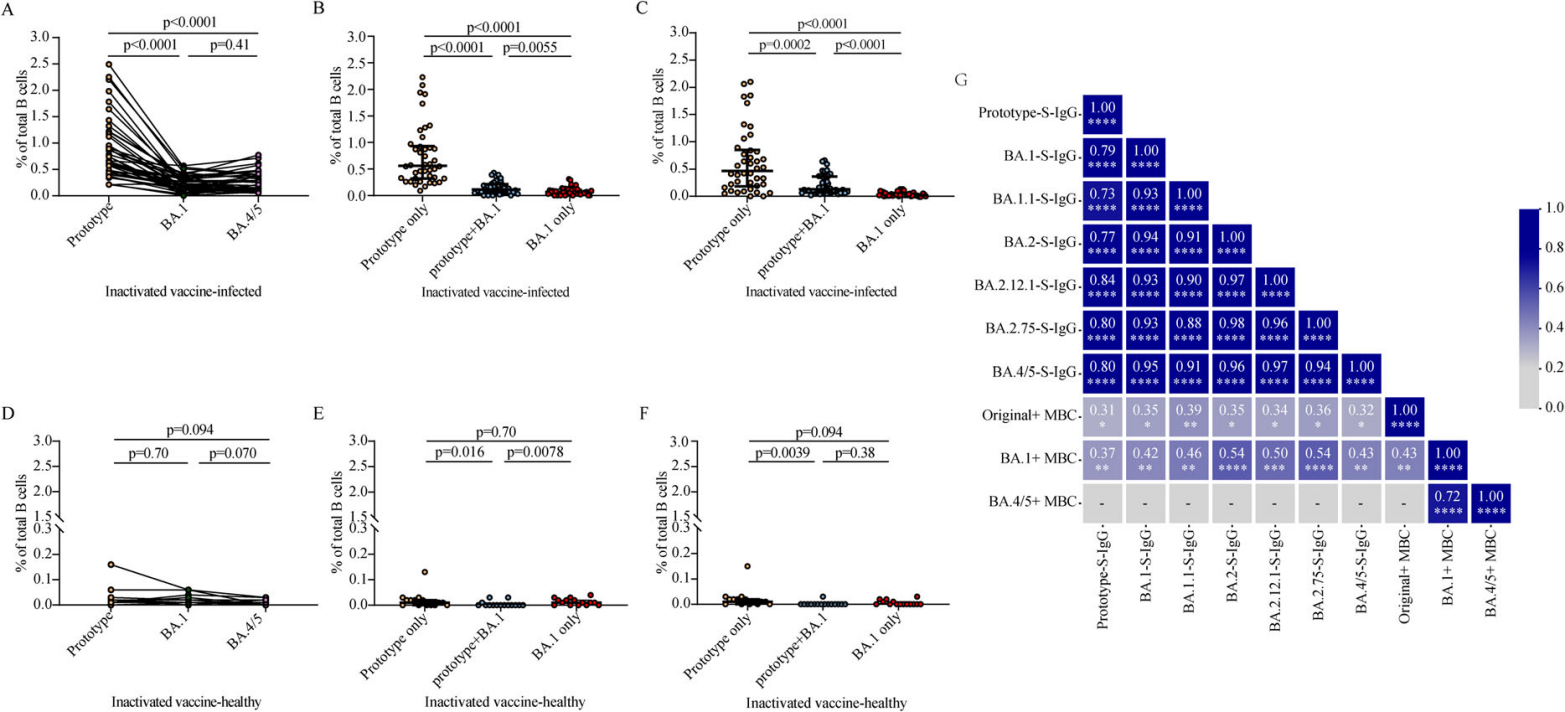
SARS-CoV-2 RBD-specific B cell responses induced by BA.1 breakthrough infection. A, D. Frequencies of memory B cells binding the prototype strain, the BA.1 strain, and the BA.4/5 strain in inactivated vaccine-infected individuals (A, n = 42) and inactivated vaccine-healthy individuals (D, n = 15). B, E. Frequencies of memory B cells binding the prototype strain only, both prototype and BA.1 stains (prototype + BA.1), and the BA.1 strain only in inactivated vaccine-infected individuals (B, n = 42) and inactivated vaccine-healthy individuals (E, n = 15). C, F. Frequencies of memory B cells binding the prototype strain only, both prototype and BA.4/5 strains (prototype + BA.4/5), and the BA.4/5 strain only in inactivated vaccine-infected individuals (C, n = 42) and inactivated vaccine-healthy individuals (F, n = 15). G. Correlation matrix of plasma IgG and memory B cells. Solid lines in (A–F) denote the median with IRQ. Multiple comparisons of memory B cell responses were performed using the Kruskal-Wallis test. For the correlation analyses, correlograms plotting the Spearman rank correlation coefficient (r) between all paired parameters were created with the scipy package (v1.7.3) running in Spyder (5.3.3). *p < 0.05, **p < 0.01, ***p < 0.001, ****p < 0.0001.

Next, we investigated the magnitude and cross-reactivity of B cell responses against the prototype strain, BA.1 and BA.4/5 in PBMCs following BA.1 breakthrough infection. We found that BA.1 breakthrough infection induced memory B cell responses to the prototype RBD in 100% (42/42), the BA.1 RBD in 95.2% (40/42), and the BA.4/5 RBD in 100% (42/42) of tested samples (Figure 4A). The frequencies of prototype RBD-reactive IgG + memory B cells in total B cells were significantly higher than those of BA.1 RBD- and BA.4/5 RBD-reactive IgG + memory B cells (both *p* < 0.0001). Frequencies of RBD-reactive memory B cells between BA.1 and BA.4/5 were not significantly different (*p* = 0.41) (Figure 4A). In BA.1 breakthrough infection individuals, memory B cells recognizing RBD epitopes shared between the prototype and BA.1 (prototype + BA.1) and between the prototype and BA.4/5 (prototype + BA.4) were considerably higher than those recognizing BA.1 only (*p* = 0.0055; Figure 4B) and BA.4/5 only (*p* < 0.0001; Figure 4C), respectively. These data indicate that BA.1 breakthrough infection in individuals vaccinated with inactivated vaccine primarily stimulates memory B cells producing epitopes conserved in the prototype strain and variants. Three-dose inactivated vaccine booster did not enhance the frequencies of memory B cells (Figure S2).

The frequencies of RBD-reactive memory B cells against prototype strain, BA.1 and BA.4/5 were significantly lower in inactivated vaccine-healthy individuals than those in inactivated vaccine-infected individuals, respectively (all *p* < 0.0001; Figure S3A–S3C). In inactivated vaccine-healthy individuals, the frequencies of RBD-reactive memory B cells among prototype strain, BA.1 and BA.4/5 were not significantly different (*p* = 0.70, *p* = 0.094, *p* = 0.070, respectively; Figure 4D). The frequencies of memory B cell between prototype only and BA.1 only had no significantly difference (*p* = 0.70), both higher those of conserved epitopes shared between the prototype strain and BA.1 (prototype + BA.1) (*p* = 0.016, *p* = 0.0078, respectively; Figure 4E). Also, the frequencies of memory B cell between prototype only and BA.4/5 only had no significantly difference (*p* = 0.094; Figure 4F). These data indicate that in inactivated vaccine-healthy individuals, the frequencies of epitopes conserved in the prototype strain and variants were not dominant.

We further performed multiparametric analyses utilizing correlation matrixes to assess the relationship among plasma S-IgG antibody titers and frequencies of memory B cells in breakthrough infection individuals. As expected, strong correlations were observed among prototype, BA.1, BA.1.1, BA.2, BA.2.12.1, BA.2.75, and BA.4/5 S-IgG titers. All prototype, BA.1, BA.1.1, BA.2, BA.2.12.1, BA.2.75, and BA.4/5 S-IgG titers were correlated with both prototype memory B cell frequencies and BA.1 memory B cell frequencies (Figure 4G, Figure S4A–S4N). In contrast, no relationship was detected between prototype or Omicron sublineages S-IgG titers and BA.4/5 memory B cell frequencies (Figure 4G). Memory B cells frequencies between prototype and BA.1 and between BA.1 and BA.4/5 were significantly associated in inactivated vaccinees by multiple metrics (Figure 4G, Figure S4O–S4P). However, no correlations were detected between prototype memory B cell frequencies and BA.4/5 memory B cell frequencies (Figure 4G, Figure S4Q). Overall, substantial correlations were observed between multiple components of plasma antibody titers and memory B cell frequencies for the BA.1 breakthrough infection, with Omicron sublineages BA4/5 exhibiting distinct immune memory profiles.

### Cross-reactivity of memory T cells induced by BA.1 breakthrough infection

**Figure 5.**
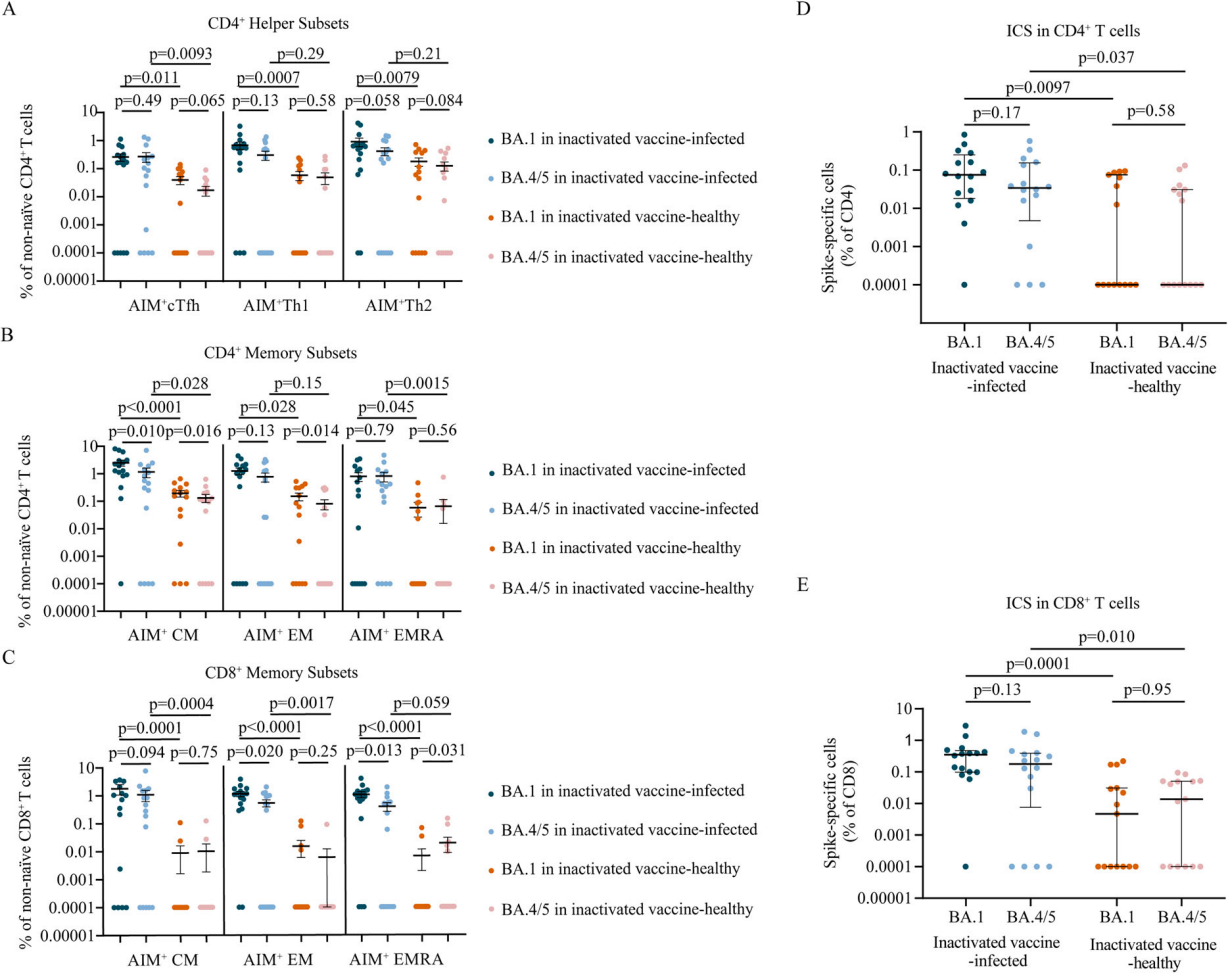
Memory T cell responses to Omicron sublineages in inactivated vaccine-infected individuals and inactivated vaccine-healthy individuals. A. Frequencies of AIM + CD4^+^ T helper subsets between BA.1 and BA.4/5 strains in inactivated vaccine-infected (n = 16) and inactivated vaccine-healthy (n = 15) individuals. B. Frequencies of AIM + CD4^+^ memory T subsets between BA.1 and BA.4/5 strains in inactivated vaccine-infected (n = 16) and inactivated vaccine-healthy (n = 15) individuals. C. Frequencies of AIM + CD8^+^ memory T subsets between BA.1 and BA.4/5 strains in inactivated vaccine-infected (n = 16) and inactivated vaccine-healthy (n = 15) individuals. D. The polyfunctional CD4^+^ T cell responses between BA.1 and BA.4/5 strains evaluated using the ICS assay in inactivated vaccine-infected (n = 16) and inactivated vaccine-healthy (n = 15) individuals. E. The polyfunctional CD8^+^ T cell responses between Omicron BA.1 and BA.4/5 strains evaluated using the ICS assay in inactivated vaccine-infected (n = 16) and inactivated vaccine-healthy (n = 15) individuals. Solid lines denote the median with IRQ. The paired comparisons of were performed using two-tailed Wilcoxon matched-pairs signed-rank test. Single comparisons between inactivated vaccine-infected and inactivated vaccine-healthy individuals were performed using the Mann-Whitney U test. cTfH = circulating T follicular helper cell; CM = central memory T cell; EM = effector memory T cell; EMRA = terminally differentiated effector memory T cell.

To determine whether breakthrough infection induced spike antigen-specific memory T cell responses, we performed flow cytometric analyses for AIM and ICS (Figure S5–S6). We found that BA.1 breakthrough infection efficiently induced spike specific CD4^+^ T cells and CD8^+^ T cells (Figure 5A–5E). BA.1 spike specific circulating T follicular helper (cTfh), Th1, and Th2 cells were detected in 68.8% (11/16), 87.5, and 87.5 (14/16) of breakthrough infection individuals, respectively. Spike-specific memory cTfh, Th1, and Th2 cell frequencies were comparable between BA.1 and BA.4/5 strain (*p* = 0.49, *p* = 0.13, *p* = 0.058, respectively; Figure 5A). Frequencies of AIM + CD4^+^ effector memory (EM) T cells (*p* = 0.13) and AIM + CD4^+^ terminally differentiated effector memory (EMRA) T cells (*p* = 0.79) were not different between BA.1 and BA.4/5, although the frequency of AIM + CD4^+^ central memory (CM) T cells for BA.4/5 was lower than that of BA.1 (*p* = 0.010) (Figure 5B). Frequencies of AIM + CD8^+^ EM T cells (*p* = 0.020) and AIM + CD8^+^ EMRA T cells (*p* = 0.013) responded to BA.4/5 RBD were lower than those responded to BA.1 RBD whereas frequencies of AIM + CD8^+^ CM T cells (*p* = 0.094) were not different significantly (Figure 5C). To access whether epitope mutations affect the functional capacity of cells, we detected the polyfunctional profiles of T cells utilizing ICS. We found that there was no significant difference in the polyfunctional CD4^+^ (*p* = 0.17) and CD8^+^ T cell (*p* = 0.13) responses between BA.1 and BA.4/5 (Figure 5D, 5E), indicating that there was no functional deficit in cross-reactive BA.1 and BA.4/5 T cell responses. Overall, the results suggest that CD4^+^ and CD8^+^ T cell responses to Omicron spike are largely preserved.

In inactivated vaccine-healthy individuals, the memory T cell responses to BA.1 and BA.4/5, including CD4^+^ helper subsets (Figure 5A), CD4^+^ memory subsets (Figure 5B), CD8^+^ memory subsets (Figure 5C), the polyfunctional profiles of CD4^+^ T cell (Figure 5D) and CD8^+^ T cell (Figure 5E), were similar with the pattern in breakthrough infection individuals. However, the polyfunctional cytokine profiles of CD4^+^ T cell (*p* = 0.0097, *p* = 0.037 for BA.1 and BA.4/5, respectively; Figure 5D) and CD8^+^ T cell (*p* = 0.0001, *p* = 0.010, respectively; Figure 5E) responses were lower in inactivated vaccine-healthy individuals than those in breakthrough infection individuals. Similar results were found in CD4^+^ helper subsets (Figure 5A), CD4^+^ memory subsets (Figure 5B), and CD8^+^ memory subsets (Figure 5C). These data indicate that the memory T cell responses to Omicron sublineages induced by inactivated vaccine-healthy individuals were lower than those elicited by breakthrough infection individuals.

## Discussion

Here, we show that BA.1 infection of individuals without vaccination does not induce effective binding antibodies, ADCC and ADCP activities, and NAbs, indicating the low immunogenic and limited ability of the BA.1 strain to stimulate strong antibody responses to Omicron sublineages. A study has shown that Omicron infection induces a limited humoral immune response in mice without vaccination [14]. These data indicate that vaccine naïve individuals infected with BA.1 remain at risk of being reinfected with SARS-CoV-2 variants. Thus, a heterologous primer-boost immunization or multivalent vaccine may benefit these Omicron infection individuals [15].

We observe that BA.1 breakthrough infection individuals show increased NAb titers against BA.1, BA.1.1, BA.2, BA.2.12.1 but only modestly increased NAb titers against BA.2.75 and BA.4/5, indicating that BA.1-boosted NAbs are poorly cross-reactive to BA.2.75 and BA.4/5. Moreover, BA.1 breakthrough infection induces very little cross-neutralization to BF.7, BQ.1, and XBB. It has been reported that BA.1 breakthrough infection of individuals immunized with COVID-19 mRNA vaccines induces strong neutralization against BA.1, BA.2 and earlier SARS-CoV-2 VOCs but not against BA.4 and BA.5 [16]. It has also been shown that NAb titers against BA.5 and BA.2.75 are lower than those against BA.1 and BA.2 in breakthrough infection individuals who had received two doses of BNT162b2 vaccine [17]. In this study, NAb titers against the Omicron variant are significantly lower than that against the prototype strain in breakthrough infection, similar with the results in natural infection and vaccinees [18–20]. These data indicate that Omicron breakthrough infection does not elicit high titers of pan-sarbecovirus NAbs, which may be due to further genetic and antigenic distance between the prototype strain and Omicron sublineages [15]. Thus, Omicron breakthrough infection cannot effectively protect individuals against the subsequent infection of BA.4/5, BA.2.75, BF.7, BQ.1, or XBB. With the rapid evolution of SARS-CoV-2, other Omicron sublineages and SARS-CoV-2 variants with even more effective immune escape capability than BA.4/5, BF.7, BQ.1, and XBB may emerge. It is urgent to develop more effective vaccines against pan-sarbecovirus to control possible high incidence of breakthrough infection and reinfections with the Omicron sublineages and future SARS-CoV-2 variants.

Fc effector functions, such as ADCC, ADCP, antibody-dependent complement deposition (ADCD), antibody-dependent cellular trogocytosis (ADCT), are important in disease control and vaccine efficacy against viral diseases [21]. During ADCC, the IgG Fc primarily binds to surface receptor FcγRIIIA expressed on NK cells and macrophages, while the Fab fragment binds to pathogen antigen on infected cells. NK cells and macrophages are activated after multimeric crosslinking of the Fc receptors and release granzymes and perforin to destruct the infected cells. During ADCP, the IgG Fc binds FcγRIIA expressed on phagocytes. Infected cells or viral particles were uptake by phagocytes through antibody opsonize, resulting in the pathogen degradation [21]. In ADCC and ADCP activities, IgG3 has the strongest binding affinity to FcγRIIIA and FcγRIIA, followed by IgG1 [22]. Collaboration of neutralization with Fc effector function may be critically important to provide highly effective humoral immunity in protection against virus infection. Neutralization may play a lesser role in controlling virus once infection has been established. Instead, Fc effector functions play an important role in the clearance of virus and virus-infected cells [23]. It has been reported that non-survival COVID-19 patients showed compromised SARS-CoV-2 RBD-specific monocyte ADCP activities [23]. We show that inactivated vaccine enhances BA.1 infection-elicited ADCC and ADCP activities against BA.1, BA.1.1, BA.2. BA.2.12.1, and BA.2.75, but not BA.4/5. The coordinate efforts of ADCC and ADCP activities and neutralization are critically needed for patients’ recovery.

Pre-existing B cell memory is critical in driving a recall response upon re-exposure to SARS-CoV-2 antigen [24]. Here, we demonstrate that BA.1 breakthrough infection induces memory B cell responses to the prototype strain and Omicron sublineages in vaccinees of inactivated vaccine. Omicron sublineage cross-reactive memory B cells display biased reactivity toward the prototype strain, suggesting that the memory B cells are established after vaccination. After re-exposure, the memory B cells re-enter germinal centers (GCs) and undergo further affinity maturation [24, 25], and obtain the capacity to recognize and respond to the Omicron variant. These cross-reactive memory B cells in BA.1 breakthrough infection display enhanced binding antibody, ADCC- and ADCP-mediating antibody, and NAb titers to Omicron sublineages. However, our data also suggest that the frequencies of memory B cells against the prototype strain are significantly higher than those of Omicron cross-reactive memory B cells, which mainly recognize conserved epitopes between prototype and variant. Thus, cross-reactive memory B cells against Omicron variant seem to be dominated by a recall of vaccine-induced immunological memory with the capacity to recognize Omicron. However, this does not exclude an ongoing clonal evolution, which can increase the response magnitude and breadth of the B cell response following BA.1 breakthrough infection. Studies have shown that somatic hypermutations generate virus-specific NAbs with increased affinity to homologous virus and emerging heterologous variants after natural infection and mRNA vaccination [26–29].

T cell response is critical for immune protection against SARS-CoV-2 infection, especially when antibodies titers wane and VOC emerge [13, 30]. Here, we show that memory T cell recall responses are generated upon BA.1 breakthrough infection. The difference in AIM between BA.1 and BA.4/5 probably is due to those mutations in particular T epitopes affect the binding to their cognate human leukocyte antigen (HLA) alleles or the binding of peptide/MHC complex to T cell receptor. Further study is needed to define specific HLA alleles and T cell epitopes linked to loss of T cell recognition. However, ICS results do not show a functional deficit in cross-reactive BA.1 and BA.4/5 T cell responses, indicating that T cell responses of Omicron spike is largely preserved. The longer-term durability of SARS-CoV-2-specific T cells in BA.1 breakthrough infection in particular memory T cell responses against none-spike proteins remain to be determined.

Our study has several limitations. First, we did not obtain consecutive samples. Longitudinal data from cohorts will help to further characterize the humoral and cellular immunities in individuals recovered from breakthrough infection. Second, the sample sizes are small, further research with more samples are necessary.

In summary, we have evaluated adaptive immune responses in BA.1 breakthrough infection individuals who received inactivated vaccine. Our study shows that BA.1 breakthrough infection induces poorly cross-neutralization against BA.2.75 and BA.4/5 and does not induce cross-neutralization to BF.7, BQ.1, and XBB. Omicron sublineages memory B cells and memory T cells are elicited in BA.1 breakthrough infection individuals. These data have important implications for understanding the vaccine effectiveness and adapted immunity to breakthrough infection in individuals fully immunized with inactivated vaccine. The Omicron sublineages, especially for those emerged after BA.4/5 strain, evade the NAb responses induced by BA.1 breakthrough infection. It is urgent to optimize the vaccine design and formulations to Omicron sublineages and future SARS-CoV-2 variants.

## Supplementary Material

Supplemental Material
