# Supplementary material for: Omicron BA.1 breakthrough infections in inactivated COVID-19 vaccine recipients induced distinct pattern of antibody and T cell responses to different Omicron sublineages
Source: Emerg Microbes Infect. 2023 May 1;12(1):2202263. doi: 10.1080/22221751.2023.2202263 (PMC10155635; doi:10.1080/22221751.2023.2202263)
Supplement: Supplemental Material [file TEMI_A_2202263_SM9028.docx]

**Omicron BA.1 breakthrough infections in inactivated COVID-19 vaccine recipients induced distinct pattern of antibody and T cell responses to different Omicron sublineages**

Li Guo^1,2,3*^, Qiao Zhang^1*^, Jingchuan Zhong^1*^, Lan Chen^1*^, Wentao Jiang^4,5,6*^， Tingxuan Huang^1,7^, Yanan Li^1^, Yin Zhang^1,7^, Liuhui Xu^1^, Xinming Wang^1^,Yan Xiao^1^, Ying Wang^1^, Xiaojing Dong^1^, Tao Dong^8,9^, Yanchun Peng^8,9^, Biao Zhang^10,11^, Yan Xie^4,5,6^, Hongmei Gao^12^ , Zhongyang Shen^4,6,13^✉, Lili Ren^1,3^†, Tao Cheng^10,11^†, Jianwei Wang^1,3^†

1 National Health Commission Key Laboratory of Systems Biology of Pathogens and Christophe Mérieux Laboratory, Institute of Pathogen Biology, Chinese Academy of Medical Sciences & Peking Union Medical College, Beijing, China

2 Haihe Laboratory of Cell Ecosystem, Tianjin, China

3 Key Laboratory of Respiratory Disease Pathogenomics, Chinese Academy of Medical Sciences, Beijing, China

4 Organ Transplant Center, Tianjin First Center Hospital, Tianjin, China

5 Laboratory of Molecular and Treatment of Liver Cancer, Tianjin First Center Hospital, Tianjin, China

6 Research Institute of Transplant Medicine, Nankai University, Tianjin, China

7 Department of Respiratory and Critical Care Medicine, West China Hospital, Sichuan University, Chengdu, Sichuan, China

8 Chinese Academy of Medical Sciences Oxford Institute, Nuffield Department of Medicine

9 MRC Human Immunology Unit, MRC Weatherall Institute of Medicine, Oxford University

10 Haihe Laboratory of Cell Ecosystem, State Key Laboratory of Experimental Hematology, National Clinical Research Center for Blood Diseases, Institute of Hematology & Blood Diseases Hospital, Chinese Academy of Medical Sciences & Peking Union Medical College, Tianjin, China

11 Tianjin Institutes of Health Science, Tianjin, China.

12 Intensive Care Unit, Emergency Medical Research Institute, Tianjin First Center Hospital, Tianjin, China

13 NHC Key Laboratory for Critical Care Medicine, Tianjin First Center Hospital, Tianjin, China;

**Table S1. Reagents used in this study**

| **Reagent** | **Information** | **Source** | **Identifier** |
| --- | --- | --- | --- |
| **Recombinant proteins** | **Position** |  |  |
| SARS-CoV-2 S protein, His Tag, Super stable trimer | Val 16-Pro1213 | ACROBiosystems | SPN-C52H9 |
| SARS-CoV-2 Spike Trimer, His Tag (BA.1/Omicron) | Val 16 - Pro 1213 | ACROBiosystems | SPN-C522a |
| SARS-CoV-2 Spike Trimer, His Tag (BA.1.1/Omicron) | Val 16 - Pro 1213 | ACROBiosystems | SPN-C5224 |
| SARS-CoV-2 Spike Trimer, His Tag (BA.2/Omicron) | Val 16 - Pro 1213 | ACROBiosystems | SPN-C5223 |
| SARS-CoV-2 Spike Trimer, His Tag (BA.2.12.1/Omicron) | Val 16 - Pro 1213 | ACROBiosystems | SPN-C522d |
| SARS-CoV-2 Spike Trimer, His Tag (BA.4/5 Omicron) | Val 16 - Pro 1213 | ACROBiosystems | SPN-C5229 |
| SARS-CoV-2 Spike Trimer, His Tag (BA.2.75/Omicron) | Val 16 - Pro 1213 | ACROBiosystems | SPN-C522f/ |
| Biotinylated SARS-CoV-2 S protein, His,Avitag™, Super stable trimer | Val 16 - Pro 1213 | ACROBiosystems | SPN-C82E9 |
| Biotinylated SARS-CoV-2 Spike RBD, His,Avitag™ | Arg 319 - Lys 537 | ACROBiosystems | SPD-C82E9 |
| Biotinylated SARS-CoV-2 Spike RBD (K417N, L452R, T478K), His,Avitag | Arg 319 - Lys 537 | ACROBiosystems | SPD-C82Eg |
| Biotinylated SARS-CoV-2 Spike RBD, His,Avitag™ (B.1.1.529/Omicron) | Arg 319 - Lys 537 | ACROBiosystems | SPD-C82E4 |
| **Antibodies** | **Clone** |  |  |
| Horseradish peroxidase conjugated goat rabbit anti-human α chain specific polyclonal IgA | NA | Sigma Aldrich | A0295 |
| Horseradish peroxidase conjugated goat anti-human Fc specific polyclonal IgG | NA | Sigma Aldrich | A1070 |
| APC-anti-human CD19 | SJ25C1 | Biolegend | 363006 |
| BV421-anti-human IgD | IA6-2 | Biolegend | 348226 |
| PerCP-Cy5.5-anti-human IgG | M1310G05 | Biolegend | 410710 |
| BV 510™ anti-human CD27 | O323 | Biolegend | 302836 |
| PE/Cy7 anti-human CD38 | HIT2 | Biolegend | 303516 |
| PerCP/Cy5.5- anti-human CD3 | SK7 | Biolegend | 344808 |
| BV650-anti-human CD4 | RPA-T4 | Biolegend | 300536 |
| PE/Cy7-anti-human CD8 | SK1 | Biolegend | 344712 |
| BV421-anti-human IFNγ | 4S.B3 | Biolegend | 502532 |
| BV711-anti-human TNFα | MAb11 | Biolegend | 502940 |
| APC-anti-human IL-2 | MQ1-17H12 | Biolegend | 500310 |
| FITC-anti-human CD107a | H4A3 | Biolegend | 328606 |
| APC anti-OX40 | Ber-Act35 | Biolegend | 350008 |
| BV421 anti-4-1BB | 4b4-1 | Biolegend | 309820 |
| PE anti-CD40L | 24-31 | Biolegend | 310806 |
| PE-Dazzle 594 anti-CCR7 | G043H7 | Biolegend | 353236 |
| BV605 anti-CXCR3 | G025H7 | Biolegend | 353728 |
| APC-Cy7 anti-CXCR5 | J252D4 | Biolegend | 356926 |
| BV785 anti-CD45RA | HI100 | Biolegend | 304140 |
| anti-CD40 | HB14 | Biolegend | 313002 |
|  |  |  |  |
| **streptavidin (SA)–fluorophore conjugates** |  |  |  |
| Streptavidin-PE | NA | Biolegend | 405204 |
| Streptavidin-BV605 | NA | Biolegend | 405229 |
| Streptavidin-BV785 | NA | Biolegend | 405249 |
| Streptavidin-BVFITC | NA | Biolegend | 405201 |
|  |  |  |  |
| **Live/Dead buffer** |  |  |  |
| Zombie NIR™ Fixable Viability Kit | NA | Biolegend | 423106 |
| BD Horizon Fixable Viability Stain 510 | NA | BD Biosciences | 564406 |
|  |  |  |  |
| **Stimulates** | **Clone** |  |  |
| Purified NA/LE Mouse Anti-Human CD3 | OKT3 | BD Biosciences | 555336 |
| Purified mouse anti-human CD28 | CD28.2 | Biolegend | 302902 |
| Purified anti-human CD49d | 9F10 | Biolegend | 304302 |
| Brefeldin A Solution (1,000X) | NA | Biolegend | 420601 |
| Monensin Solution (1,000X) | NA | Biolegend | 420701 |
| PMA/Ionomycin Mixture (250×) | NA | Multi Science | CS1001 |
| Hunan Interleukin-2 | NA | PeproTech | 200-02 |
|  |  |  |  |
| Human TruStain FcX™ (Fc Receptor Blocking Solution) | NA | Biolegend | 422302 |
| Foxp3 / Transcription Factor Staining Buffer Set | NA | Invitrogen | 00-5523-00 |

**Table S2. The summary information of participants for antibody and immune cell responses analyses**

| **Item** | **Samples** | **Detection methods** | **Sample No.**  **(Breakthrough infection)** | **Sample No. (Vaccine naïve-infected)** | **Sample No. (Inactivated vaccine-healthy)** | **Sample No. (Vaccine naïve-healthy)** |
| --- | --- | --- | --- | --- | --- | --- |
| Mucosal sIgA | TS | ELISA | 55 | 5 | 0 | 0 |
| S-IgG | Plasma | ELISA | 55 | 5 | 15 | 15 |
| ADCC antibodies, and ADCP antibody | Plasma | ADCC Bioassay, ADCP Bioassay | 55 | 5 | 15 | 15 |
| Neutralizing antibody | Plasma | MN, PVMN | 55 | 5 | 15 | 15 |
| Memory B cell responses | PBMC | Flow cytometric assay | 42 | 0 | 15 | 0 |
| Memory T cell responses | PBMC | AIM, ICS | 16 | 0 | 15 | 0 |

TS=throat swab; sIgA=secretory IgA; ELISA=Enzyme-linked immunosorbent assay; ADCC=antibody-dependent cellular cytotoxicity; ADCP=antibody-dependent Cellular Phagocytosis; MN=Microneutralization assay; PVMN=Pseudovirus microneutralization assays; AIM=Activation induced marker assay; ICS=Intracellular cytokine staining; Vaccine naïve= without receive COVID-19 inactivated vaccine.

Figure S1. Gating strategy for SARS-CoV-2-specific memory B cells


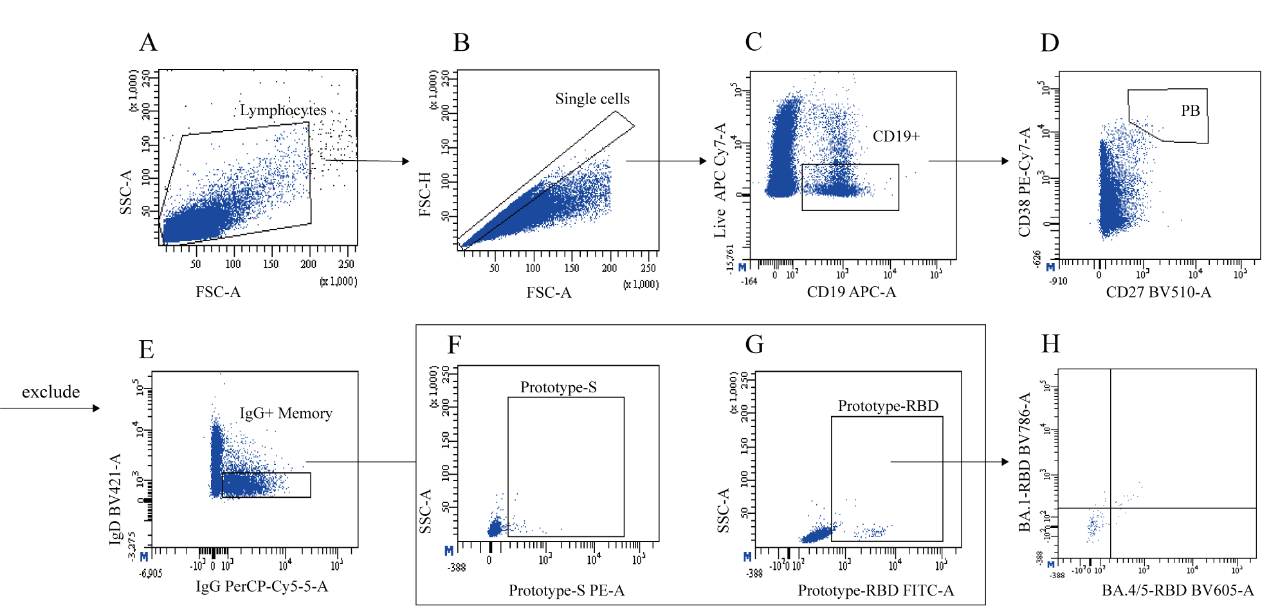


Total B cells were enriched using Pan B Cell Isolation Kit from PBMC samples prior to staining. **A–C**. Live CD19^+^ B cells were identified. **D**. Plasmablasts (PB) were then identified as CD27^+^ CD38^+^ CD19^+^ B cells and were excluded for downstream analysis. **E**. IgG+ memory B cells were identified based on IgD^-^IgG^+^ B cells. **F–G**. Prototype spike+ memory cells and RBD+ memory B cells were then analyzed for binding to prototype spike probes and RBD probe, respectively. **H**. Prototype RBD-specific memory B cells were subsequently analyzed for binding to prototype RBD and co-binding to a panel of variant RBD probes, including BA.1 and BA.4/5 RBDs.

Figure S2. SARS-CoV-2 RBD-specific B cell responses induced by BA.1 breakthrough infection


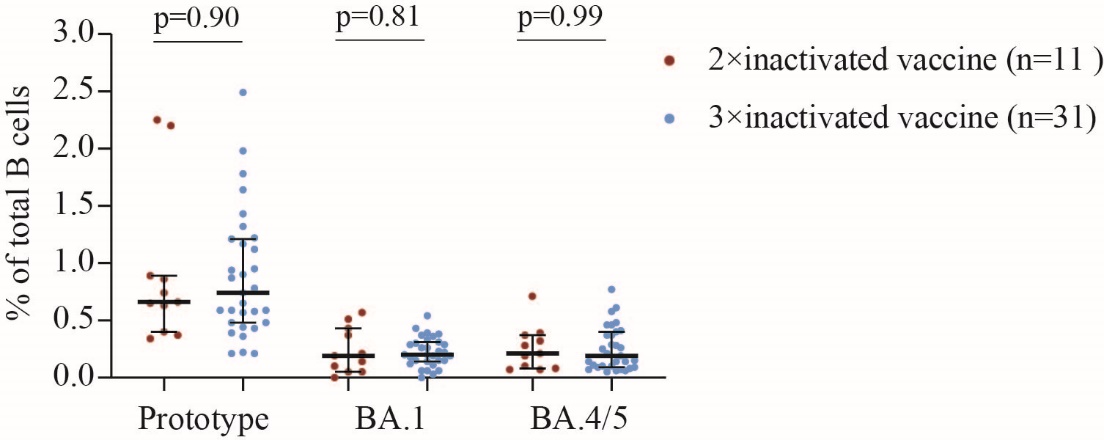


SARS-CoV-2 RBD-specific B cell responses induced by BA.1 breakthrough infection in individuals with 2-dose inactivated vaccine primary immunization, and 3-dose inactivated vaccine booster. The solid lines denote the median with interquartile range (IRQ). The comparisons of memory B cells frequencies were performed using Mann-Whitney U test.

Figure S3. SARS-CoV-2 RBD-specific B cell responses induced by inactivated vaccine-infected and inactivated vaccine-healthy individuals.


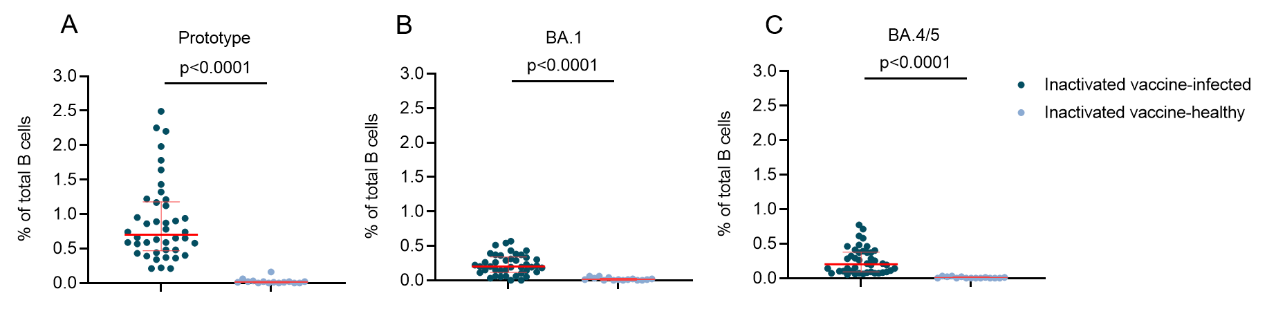


SARS-CoV-2 RBD-specific B cell responses against prototype (A), BA.1 (B), and BA.4/5 (C) strain induced by inactivated vaccine-infected (n=42) and inactivated vaccine-healthy individuals (n=15). The solid lines denote the median with interquartile range (IRQ). The comparisons of memory B cells frequencies were performed using Mann-Whitney U test.

Figure S4. Correlation analyses of IgG and memory B cells


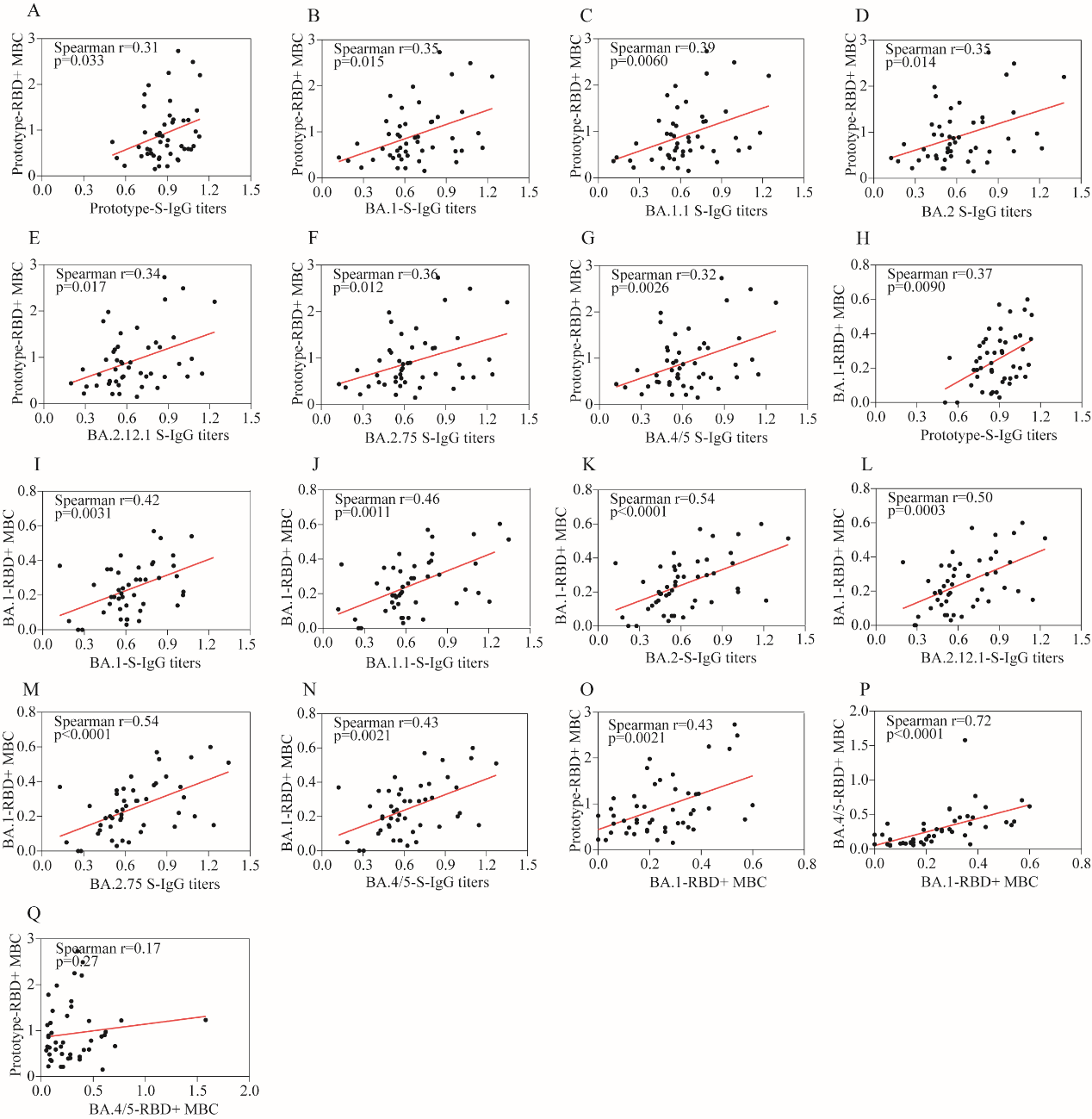


**A–G**. The correlation between prototype memory B cells and S-IgG titers against prototype (A), BA.1 (B), BA.1.1 (C), BA.2 (D), BA.2.12.1 (E), BA.2.75 (F), BA.4/5(G). **H–N.** The correlation between Omicron BA.1 memory B cells and S-IgG titers against prototype (H), BA.1 (I), BA.1.1 (J), BA.2 (K), BA.2.12.1 (L), BA.2.75 (M), BA.4/5(N). **O.** The correlation between prototype memory B cells and Omicron BA.1 memory B cells. **P**. The correlation between Omicron BA.1 memory B cells and Omicron BA.4/5 memory B cells. **Q**. The correlation between prototype memory B cells and Omicron BA.4/5 memory B cells. Spearman correlation analysis was performed for the correlation analyses. Figure S5. Gating strategy for AIM+ memory T cell analysis


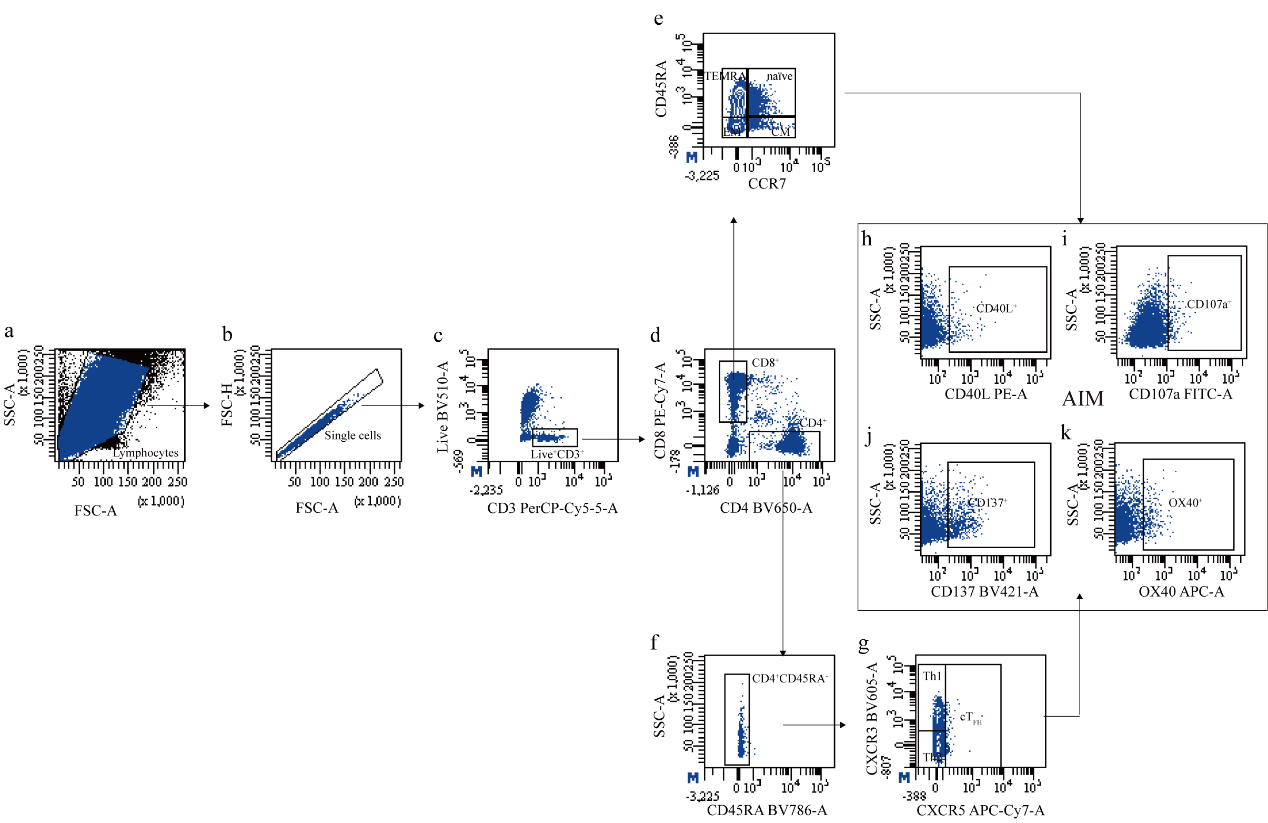


**A–D**. Gating for CD4^+^/CD8^+^ T cells. Cells were gated on single cells by a forward side scatter gate, followed by CD3/CD4/CD8 gating excluding dead cells, CD14^+^, CD19^+^, and CD16^+^ cells. **E**. Memory T cell subsets were identified based on surface expression of CD45RA, CD27, and CCR7. **F–G**. CD4^+^ T helper subsets were defined based on chemokine receptor CXCR3 and CXCR5 expression. **H-K**. T cell express the activation induced markers: CD40L, CD107a, CD137, and OX40. AIM+ T cells were measured as a percentage of coexpression at least two of four markers (CD40L, CD107a, CD137, and OX40) after stimulation of PBMCs with the Spike peptide pool.

Figure S6. Gating strategy of memory T cell for cytokine-producing analysis after stimulation with peptide pools.


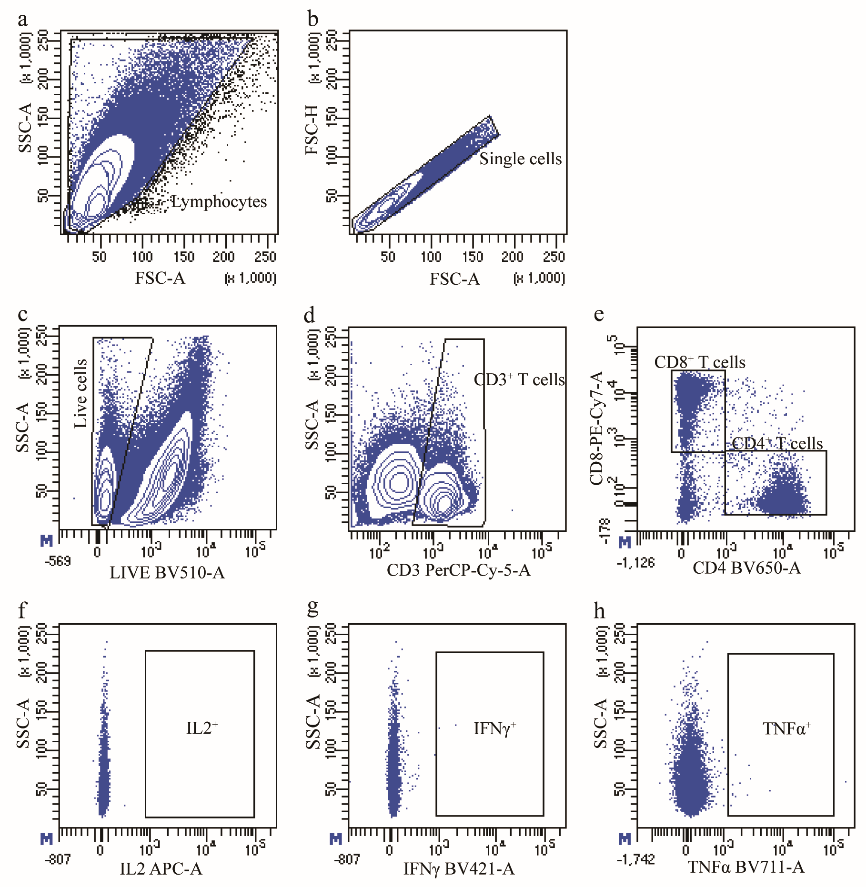


**A–E**. Gating for CD4^+^/CD8^+^ T cells. Cells were gated on single cells by a forward side scatter gate, followed by CD3/ CD4/CD8 gating to exclude dead cells, CD14^+^, CD19^+^, and CD16^+^ cells. **F–H** Gating for IL-2+/-, IFN-γ+/-, and TNF-α+/-. Population was based on corresponding negative controls. T cell responses were expressed as proportion of IL-2, IFNγ, and TNFα produced by SARS-CoV-2-specific CD4 and CD8 T cells after stimulation of PBMCs with the Spike peptide pool.
